# Regulator of G protein signaling 2 as a suppressor of sphingosine-1-phosphate 2– and 3–mediated signaling in colon cancer cells

**DOI:** 10.1016/j.jbc.2025.110554

**Published:** 2025-08-05

**Authors:** Jaejun Eo, Nahyun Kim, Sungho Ghil

**Affiliations:** Department of Life Science, Kyonggi University, Suwon, Republic of Korea

**Keywords:** bioluminescence resonance energy transfer, Gα subunits, membrane translocation, tumor progression

## Abstract

Sphingosine-1-phosphate receptor 2 and 3 (S1P_2_ and S1P_3_) are G protein–coupled receptors that mediate extracellular sphingosine-1-phosphate (S1P) signaling into cells. S1P_2_ and S1P_3_ are highly expressed in colon cancer cells, but their roles in cancer progression–related cellular phenotypes are not well understood. Recent studies suggest that regulator of G protein signaling 2 (RGS2) interacts with G protein–coupled receptors, either directly or indirectly, to regulate their signaling. However, the precise role of RGS2 in S1P_2_ and S1P_3_ signaling remains uninvestigated. In this study, we examined the interaction of RGS2 with S1P_2_ and S1P_3_ using bioluminescence resonance energy transfer analysis and assessed its impact on S1P_2_- and S1P_3_-mediated signaling in 293T and HCT116 cells. Bioluminescence resonance energy transfer analysis revealed that RGS2 and Gα subunits simultaneously bind to S1P_2_ and S1P_3_. Furthermore, in the presence of these receptors, RGS2 translocated from the cytoplasm to the cell membrane. These interactions and membrane translocation were not observed with the RGS1 negative control, highlighting the specificity of RGS2 for S1P_2_ and S1P_3_. RGS2 expression inhibited the activation of Gαi, Gαq, and Gα12, key signaling pathways mediated by S1P_2_ and S1P_3_. S1P_2_ and S1P_3_ activation significantly enhanced cell migration and the expression of cancer-associated genes, effects that were effectively suppressed by RGS2 expression. In contrast, RGS1 failed to inhibit S1P_2_- and S1P_3_-mediated Gα signaling as well as downstream effects, such as enhanced cell migration and cancer-associated gene expression. Our findings show that RGS2 suppresses S1P2- and S1P3-mediated cancer-associated cellular phenotypes by interacting with these receptors and inhibiting Gα-mediated signaling.

G protein–coupled receptors (GPCRs) are seven-transmembrane domain proteins that regulate a wide range of intracellular signaling pathways in response to external stimuli, such as hormones and neurotransmitters. These receptors play a vital role in physiological processes and are implicated in various diseases. Signal transduction through GPCRs depends on the activation of heterotrimeric G proteins, which consist of three subunits: Gα, Gβ, and Gγ. The Gα subunit is further classified into four primary families—Gαs, Gαi/o, Gαq, and Gα12/13—based on sequence homology (1, 2). When an agonist binds to a GPCR, the GDP bound to the Gα subunit is replaced with GTP, causing the heterotrimer to dissociate into Gα and Gβγ subunits. Each subunit then activates distinct signaling pathways. G protein signaling is terminated when the intrinsic GTPase activity of Gα hydrolyzes GTP back to GDP, restoring the inactive heterotrimeric state (3).

Sphingosine-1-phosphate (S1P) is a bioactive lysosphingolipid that regulates various cellular processes, including proliferation, migration, apoptosis, and invasion. S1P exerts its effects through five distinct GPCR subtypes, designated S1P1 to S1P5 (4). The S1P_2_ receptor, coupled with Gαi/o, Gαq/11, and Gα12/13 (5), is localized to both the plasma membrane and cytoplasm and is expressed in the central nervous and immune systems, where it plays crucial roles in apoptosis, cell proliferation, and actin remodeling (6). In colon cancer patients, S1P_2_ has been shown to undergo internalization, leading to increased intracellular calcium levels and contributing to resistance against 5-fluorouracil, a widely used chemotherapeutic agent. In addition, studies in dextran sulfate sodium-induced colitis models have demonstrated that blocking the S1P_2_–Gα12/13 signaling pathway, specifically the RhoA–ROCK axis, exacerbates inflammatory bowel disease (7, 8). S1P_3_ mediates cellular processes, such as proliferation, differentiation, and migration, similar to S1P_2_. It transduces signals through G proteins, including Gαi/o, Gαq, and Gα12/13, to regulate downstream effectors, such as VEGF-A, Rho, PI3K, PLC, and Ras (4, 9). Elevated expression of S1P_3_ has been observed in colon cancer patients, and this overexpression is associated with poor prognosis (10).

Regulator of G protein signaling (RGS) proteins are essential modulators of GPCR signaling, acting as GTPase-activating proteins to enhance the hydrolysis of GTP on the Gα subunit. This process accelerates the conversion of GTP to GDP, effectively terminating the signaling cascade. Given the central role of GPCRs as therapeutic targets, elucidating the physiological functions of RGS proteins is crucial for advancing drug development strategies (11, 12). RGS2, in particular, has been shown to significantly modulate signaling pathways by inhibiting phospholipase C activation associated with Gαq-coupled receptors as well as by suppressing intracellular Ca^2+^ mobilization and ion channel activation (13).

Several studies have demonstrated that RGS2 directly or indirectly interacts with GPCRs, leading to the suppression of GPCR-mediated downstream signaling (14). We previously demonstrated that RGS2 interacts with protease-activated receptors (PARs) and G protein-coupled receptor 55 (GPR55) to inhibit their associated signaling pathways (15, 16, 17). However, the specific interactions between RGS2 and S1P_2_ or S1P_3_, as well as the effects of RGS2 on S1P_2_- and S1P_3_-mediated signaling, remain largely unexplored.

In this study, we used bioluminescence resonance energy transfer (BRET) analysis to investigate the interactions between S1P receptors and RGS proteins in living cells. We further analyzed the subcellular localization of RGS2 and its associated S1P receptors to elucidate their spatial dynamics. In addition, we examined the inhibitory effects of RGS2 on Gα signaling induced by S1P_2_ and S1P_3_ activation. Finally, we evaluated whether RGS2 expression affects S1P_2_- and S1P_3_-mediated cancer–associated cellular phenotypes by assessing cell migration and the expression of cancer-related genes in HCT116 colon cancer cells.

## Results

### Specific interaction of RGS2 with S1P_2_ and S1P_3_ in live cells

**Figure 1:**
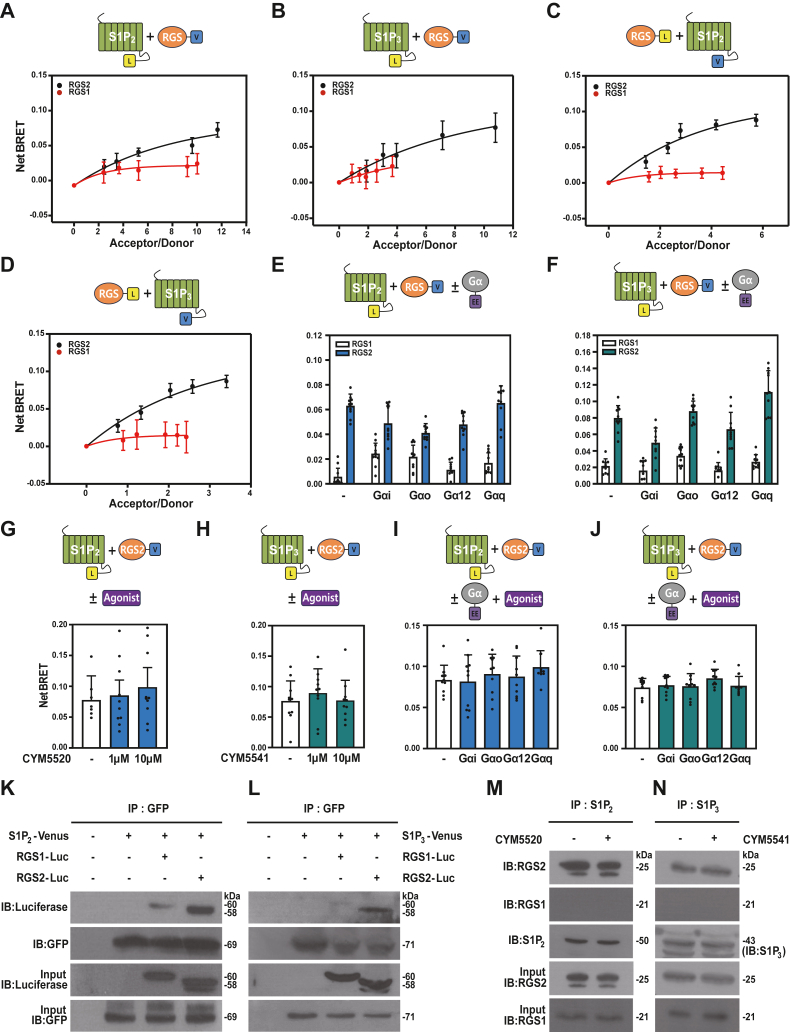
**Specific interaction of S1P2 and S1P3 with RGS2 in living cells.***A*–*J*, schematic representation of proteins and agonists used in the BRET analysis. *A* and *B*, BRET analysis was performed in 293T cells transfected with varying amounts of RGS1-Venus (*red*) or RGS2-Venus (*black*) (0, 0.5, 0.75, 1.0, 1.5, and 2.0 μg) and a constant amount of S1P_2_-Luc (0.03 μg) (*A*) or S1P_3_-Luc (0.03 μg) (*B*), as indicated. *C* and *D*, BRET analysis was conducted in 293T cells transfected with a constant amount of RGS1-Luc (*red*) or RGS2-Luc (*black*) (0.03 μg) and varying amounts of S1P_2_-Venus (0, 0.5, 0.75, 1.0, 1.5, and 2.0 μg) (*C*) or S1P_3_-Venus (*D*), as indicated. *E* and *F*, BRET analysis in 293T cells was conducted with cotransfection of RGS1-Venus (2.0 μg), RGS2-Venus (2.0 μg), Gα isoforms (Gαi^EE^, Gαo^EE^, Gαq^EE^, or Gα12^EE^) (0.5 μg), and either S1P_2_-Luc (0.03 μg) (*E*) or S1P_3_-Luc (0.03 μg) (*F*), as indicated. *G* and *H*, BRET analysis was conducted in 293T cells cotransfected with RGS2-Venus (2.0 μg) and either S1P_2_-Luc (0.03 μg) (g) or S1P_3_-Luc (0.03 μg) (*H*), as indicated, in the presence or the absence of specific agonists (CYM5520 for S1P_2_ and CYM5541 for S1P_3_). *I* and *J*, BRET analysis was performed in 293T cells transfected with RGS2-Venus (2.0 μg), S1P_2_-Luc (0.03 μg) (*I*), S1P_3_-Luc (0.03 μg) (*J*), and Gα isoforms (Gαi^EE^, Gαo^EE^, Gαq^EE^, or Gα12^EE^) (0.5 μg), followed by treatment with specific agonists. *K* and *L*, coimmunoprecipitation analysis was performed in 293T cells cotransfected with RGS1-Luc or RGS2-Luc (10 μg), together with either S1P2-Venus (5.0 μg) (*K*) or S1P3-Luc (5.0 μg) (*L*), as indicated. Immunoprecipitation was conducted using an anti-GFP antibody, and immunoblotting was performed using the indicated antibodies. Ten percent of the cell lysate used for immunoprecipitation was loaded as input to confirm protein expression levels. *M* and *N*, coimmunoprecipitation analysis was also performed in HCT116 cells. Immunoprecipitation was carried out using antibodies specific to the corresponding receptors (S1P_2_ or S1P_3_), and immunoblotting was conducted with anti-RGS1 and anti-RGS2 antibodies, as indicated. Ten percent of the immunoprecipitated samples were analyzed to verify successful pulldown and detection. All experiments were conducted in triplicate with a minimum of three independent biological replicates. Data are shown as mean ± SD, and statistical significance was determined using one-way ANOVA followed by Tukey’s post hoc test. BRET, bioluminescence resonance energy transfer; RGS2, regulator of G protein signaling 2; S1P2, sphingosine-1-phosphate receptor 2; S1P3, sphingosine-1-phosphate receptor 3.

To investigate whether RGS2 forms a complex with either S1P_2_ or S1P_3_ in live cells, we performed BRET analysis using 293T cells. Cells were transfected with a fixed amount of RGS2-Venus and increasing quantities of either S1P_2_- (Fig. 1*A*) or S1P_3_-Luc (luciferase) (Fig. 1*B*) plasmids, with RGS1-Venus serving as a negative control. The netBRET values increased proportionally with acceptor expression in the presence of RGS2 but not RGS1, demonstrating that S1P_2_ and S1P_3_ specifically interact with RGS2 in live cells. To validate these findings, we repeated the BRET analysis using RGS2-Luc and increasing amounts of either S1P_2_- (Fig. 1*C*) or S1P_3_-Venus (Fig. 1*D*) plasmids. The results further confirmed that RGS2, but not RGS1, interacts with both S1P_2_ and S1P_3_.

Given that S1P_2_ and S1P_3_ are coupled with Gαi/o, Gαq/11, and Gα12/13 proteins (5), we subsequently evaluated the potential involvement of Gα proteins in the interactions between RGS2 and S1P_2_ or S1P_3_ receptors. 293T cells were cotransfected with RGS2-Venus, a glutamate–glutamate-tagged isoform of Gα (Gαi^EE^, Gαo^EE^, Gαq^EE^, or Gα12^EE^), and either S1P_2_ (Fig. 1*E*) or S1P_3_ (Fig. 1*F*). RGS1-Venus was used as a negative control. The presence of any Gα isoform had no effect on the interaction between RGS2 and S1P_2_ or S1P_3_, suggesting that these interactions occur independently of Gα subunit association. Thus, none of the tested Gα isoforms competes with RGS2 for binding to either S1P_2_ or S1P_3_. We also examined whether agonist stimulation affects the interaction of RGS2 with S1P_2_ and S1P_3_. BRET analysis was performed on 293T cells transfected with RGS2-Venus and either S1P_2_- or S1P_3_-Luc in the absence or the presence of specific agonists (CYM5540 or CYM5541) (Fig. 1, *G* and *H*). The interaction between RGS2 and S1P_2_ or S1P_3_ was unaffected by agonist stimulation, indicating that their binding is independent of receptor activation. This suggests that RGS2 remains bound to S1P_2_ and S1P_3_ regardless of agonist stimulation. We next investigated effect of agonist stimulation on interaction of S1P_2_- and S1P_3_-Luc with RGS2-Venus in the presence of Gα isoforms. The cells were transfected with S1P_2_-Luc, S1P_3_-Luc, RGS2-Venus, and Gα^EE^, as indicated, and performed BRET analysis in the presence of agonists (fig1Fig. 1, *I* and *J*). The interaction between RGS2 and S1P receptors is unaffected by Gα expression or receptor activation.

To address the possibility that the observed BRET signal between the receptors and RGS2 might result from bystander effects rather than a specific interaction, we performed an additional control experiment using untagged receptors. 293T cells were cotransfected with RGS2-Luc and either S1P_2_-Venus or S1P_3_-Venus, along with untagged S1P_2_ or S1P_3_ receptors, and subjected to BRET analysis (Supplementary Information 1). Coexpression of untagged receptors significantly reduced the BRET signal between the receptor and RGS2, indicating that the previously observed BRET response reflects a specific association rather than membrane proximity alone.

As an additional approach to distinguish specific interactions from nonspecific membrane effects, we employed a membrane-anchored YFP construct (Lyn-YFP) as a BRET acceptor. RGS2-Luc was coexpressed with either S1P_2/3_-Venus or Lyn-YFP in 293T cells. While BRET signals were detected between RGS2-Luc and S1P_2/3_-Venus, no BRET signal was observed between RGS2-Luc and Lyn-YFP, suggesting that membrane localization of RGS2 alone is not sufficient to induce BRET (Supplementary Information 2*A*). To further evaluate the potential contribution of bystander effects, we coexpressed untagged S1P_2_ or S1P_3_ receptors along with RGS2-Luc and Lyn-YFP in 293T cells. As shown in Supplementary Information 2*B*, no BRET signal was detected under these conditions, indicating the absence of nonspecific interactions because of receptor-mediated membrane recruitment. Notably, this result remained unchanged even after receptor stimulation. These findings collectively support the notion that the interaction between RGS2 and S1P_2/3_ is specific, rather than a consequence of membrane crowding or other bystander effects.

Our previous BRET results demonstrated that S1P_2_ and S1P_3_ specifically interact with RGS2. However, we could not exclude the possibility that this interaction might be mediated by Gα proteins. To address this issue, we generated an RGS2 mutant (RGS2^S179D^) that is deficient in Gαq binding (18, 19). Wildtype and mutant RGS2 plasmids were transfected into 293T cells, and successful expression of both constructs was confirmed by immunoblot analysis (Fig. S3*A*). To test the contribution of endogenous Gα proteins to the interaction, we cotransfected S1P receptors with either wildtype or mutant RGS2, along with plasmids encoding several Gα subunits, and performed BRET analysis. The results showed no significant difference in BRET signals between wildtype and mutant RGS2, suggesting that the interaction between RGS2 and S1P_2/3_ is not substantially dependent on endogenous Gα proteins (Fig. S3, *B* and *C*).

To further validate these findings, coimmunoprecipitation assays were performed using 293T cells transfected with RGS2-Luc and either S1P_2_- (Fig. 1*K*) or S1P_3_-Venus (Fig. 1*L*) plasmids. RGS2 was successfully detected in the immunoprecipitated complexes using GFP-specific antibodies, providing additional evidence for the interaction of RGS2 with both S1P_2_ and S1P_3_ receptors. In contrast, RGS1 exhibited no detectable interaction with these receptors. To further confirm these interactions under endogenous expression conditions, we conducted additional coimmunoprecipitation assays using HCT116 cells. Immunoprecipitation was performed using antibodies against S1P_2_ or S1P_3_, and the presence of RGS1 or RGS2 in the immunocomplexes was assessed by immunoblotting with specific antibodies against each protein. Consistent with the overexpression system, RGS2—but not RGS1—specifically interacted with both S1P_2_ and S1P_3_ receptors, further supporting the selectivity of RGS2 in a native cellular context. Notably, these interactions were observed regardless of the presence or the absence of agonist stimulation (Fig. 1, *M* and *N*). Collectively, these findings demonstrate that RGS2 specifically interacts with S1P_2_ and S1P_3_ receptors in both overexpression and endogenous settings, independent of Gα subunit expression or agonist stimulation.

### Subcellular localization of RGS2 and S1P_2_ and S1P_3_

**Figure 2:**
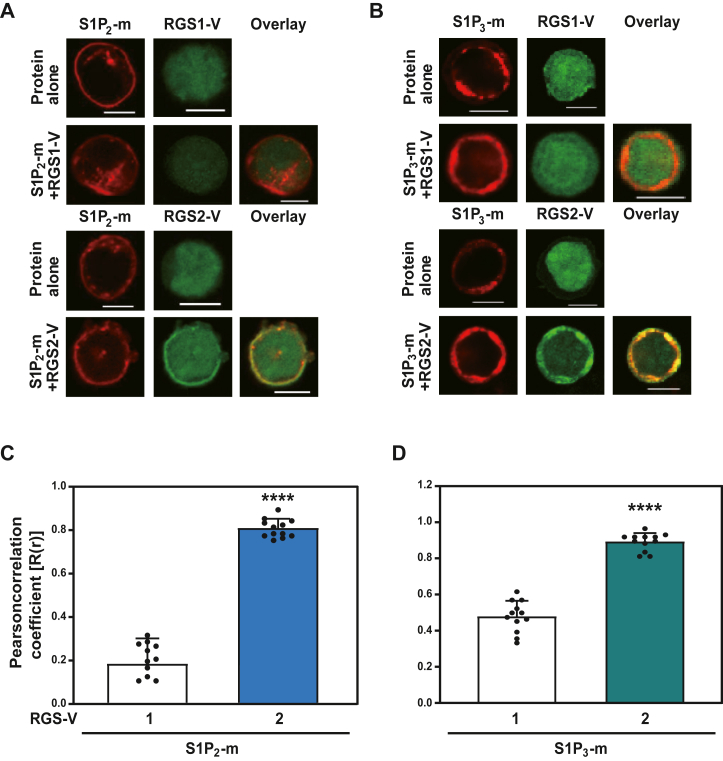
**Colocalization of S1P2, S1P3, and RGS2 expression in living cells.***A* and *B*, 293T cells were transfected with 1.0 μg each of RGS1-Venus (RGS1-V), RGS2-Venus (RGS2-V), and either S1P_2_-mCherry (S1P_2_-m) (*A*) or S1P_3_-mCherry (S1P_3_-m) (*B*), as indicated. Confocal laser microscopy was used to observe the cells. Scale bar represents 10 μm. *C* and *D*, Pearson's correlation coefficients were calculated to quantify the linear relationship between RGS2-Venus and S1P_2_-mCherry (*C*) or S1P_3_-mCherry (*D*), using RGS1-Venus as the control. Statistical significance was determined by comparing the expression of RGS1-Venus with that of RGS2-Venus (∗∗∗∗*p* < 0.001). Each experiment was repeated independently at least three times. Data are presented as mean ± SD, and statistical significance was determined using one-way ANOVA followed by Tukey’s post hoc test. RGS2, regulator of G protein signaling 2; S1P2, sphingosine-1-phosphate receptor 2; S1P3, sphingosine-1-phosphate receptor 3.

To investigate the subcellular localization of RGS proteins in the presence of S1P receptors, we cotransfected 293T cells with RGS1-Venus or RGS2-Venus along with either S1P_2_- or S1P_3_-mCherry (Fig. 2). In the absence of S1P receptors, RGS-Venus proteins were predominantly localized to the cytoplasm. However, coexpression of either S1P_2_ or S1P_3_ specifically induced the translocation of RGS2-Venus from the cytoplasm to the plasma membrane. The subcellular localization of RGS1-Venus remained unaffected by S1P receptor expression (Fig. 2, *A* and *B*). To evaluate the colocalization between S1P receptors and RGS proteins, Pearson's correlation coefficients were calculated. The correlation coefficients for RGS2 with S1P_2_ or S1P_3_ were significantly higher than those for RGS1 with S1P receptors (Fig. 2, *C* and *D*).

### Interaction of S1P_2_ and S1P_3_ with Gα and RGS2 in HCT116 cells

**Figure 3:**
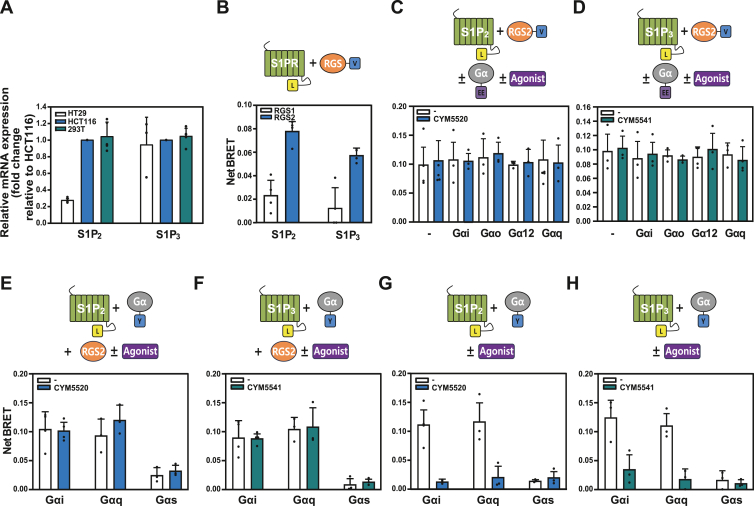
**Effect of RGS2 on S1P2- and S1P3-mediated Gα signaling.***A*, mRNA expression levels of S1P_2_ and S1P_3_ were quantified in HT29, HCT116, and 293T cells using RT–qPCR analysis. *B*, BRET analysis was conducted in HCT116 cells transfected with 2.0 μg each of RGS1-Venus and RGS2-Venus, and 0.03 μg each of S1P_2_-Luc and S1P_3_-Luc, as indicated. *C* and *D*, BRET analysis was performed in HCT116 cells cotransfected with 2.0 μg of RGS2-Venus, 0.5 μg of Gα^EE^ isoforms (Gαi^EE^, Gαo^EE^, Gα12^EE^, or Gαq^EE^), and 0.03 μg of either S1P_2_-Luc (*C*) or S1P_3_-Luc (*D*), in the absence or the presence of specific agonists (CYM5520 for S1P_2_ and CYM5541 for S1P_3_). *E* and *F*, HCT116 cells were transfected with 2.0 μg of Gα-YFP isoforms (Gαi-YFP, Gαq-YFP, or Gαs-YFP), 0.5 μg of RGS2, and 0.03 μg of either S1P_2_-Luc (*E*) or S1P_3_-Luc (*F*). BRET analysis was performed following treatment with or without specific agonists. *G* and *H*, BRET analysis was conducted in HCT116 cells transfected with 2.0 μg of Gα-YFP isoforms (Gαi-YFP, Gαq-YFP, or Gαs-YFP) and 0.03 μg of either S1P_2_-Luc (*G*) or S1P_3_-Luc (*H*), followed by treatment with or without specific agonists. All experiments were performed in triplicate with at least three independent biological replicates. Data are presented as mean ± SD, and statistical significance was determined using one-way ANOVA followed by Tukey’s post hoc test. BRET, bioluminescence resonance energy transfer; qPCR, quantitative PCR; RGS2, regulator of G protein signaling 2; S1P2, sphingosine-1-phosphate receptor 2; S1P3, sphingosine-1-phosphate receptor 3.

Previous studies have shown that S1P_2_ and S1P_3_ are highly expressed in colon cancer (20). In our study, RT–quantitative PCR (qPCR) analysis was performed to compare the expression levels of S1P_2_ and S1P_3_ between colon cancer cell line, HT29 and HCT116, and 293T cells (Fig. 3*A*). A comparative analysis of intrinsic expression levels of S1P_2_ and S1P_3_, normalized to the expression levels in HCT116 cells, revealed that HCT116 cells exhibited higher expression of S1P_2_ compared with HT29 cells, whereas the expression levels of S1P_3_ were similar between the two cell lines. Interestingly, the expression levels in 293T cells were comparable to those in HCT116 cells.

BRET analysis was performed in HCT116 cells to investigate the interactions among S1P_2/3_, RGS2, and Gα. Cells were cotransfected with RGS2-Venus and either S1P_2_-Luc or S1P_3_-Luc, and subsequent BRET analysis was conducted (Fig. 3*B*). The results demonstrated that both S1P_2_ and S1P_3_ specifically interacted with RGS2 but not with RGS1. To evaluate the Gα expression and agonist stimulation of these interactions, we coexpressed untagged Gα isoforms along with S1P_2/3_-Luc and RGS2-Venus, followed by BRET analysis in the absence or the presence of specific agonists. Consistent with the results observed in 293T cells, the interactions were found to be independent of Gα involvement and agonist stimulation (Fig. 3, *C* and *D*).

We replaced RGS2-Venus with Gα-YFP in the BRET pair and cotransfected cells with untagged RGS2 and S1P_2/3_-Luc, followed by BRET analysis in the absence or the presence of specific agonists (Fig. 3, *E* and *F*). The results showed high BRET values when Gαi-YFP or Gαq-YFP was expressed, whereas the values decreased when Gαs-YFP was expressed. Agonist stimulation did not alter these interactions.

To confirm whether agonist stimulation effectively activates receptors in our BRET system, we expressed S1P_2_-Luc, S1P_3_-Luc, and Gα-YFP, as indicated, in HCT116 cells and performed BRET analysis in the presence and the absence of agonists. The results showed that agonist stimulation reduced the interaction of S1P receptors with Gαi and Gαq, whereas Gαs, which does not couple with S1P receptors, remained unaffected.

### Specific inhibition of S1P_2_- and S1P_3_-mediated Gα signaling by RGS2

**Figure 4:**
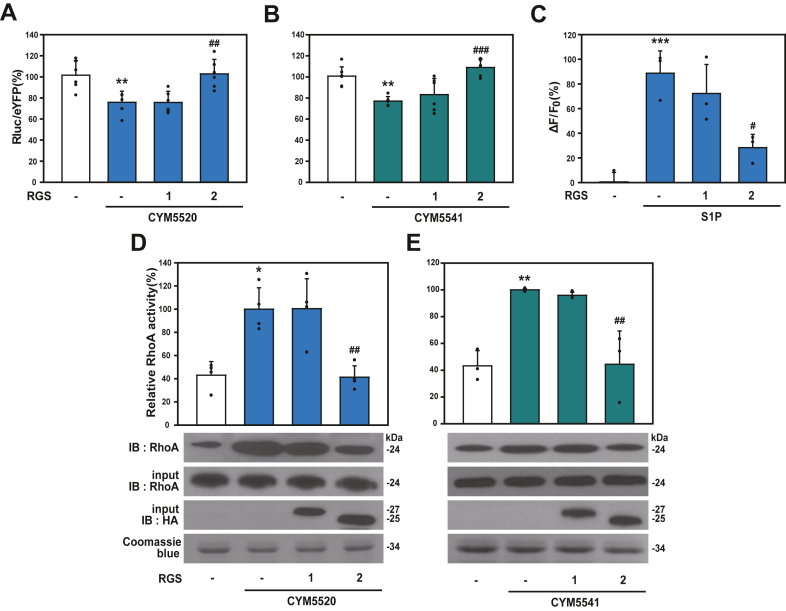
**Effect of RGS2 on S1P2 and S1P3-Gα-mediated signaling in cells.***A* and *B*, HCT116-CAMYEL cells, stably expressing the CAMYEL plasmid, were transfected with 2.0 μg of RGS1-HA or RGS2-HA, as indicated. Cells were treated with 1 μM of either the S1P_2_-specific agonist CYM5520 (*A*) or the S1P_3_-specific agonist CYM5541 (*B*), and luciferase activity was measured to assess intracellular cAMP levels. *C*, 293T cells were transfected with 0.2 μg of RGS1-HA or RGS2-HA. After incubation with Fluo-4 dye-loading solution, cells were treated with 30 μM S1P, an agonist for both S1P_2_ and S1P_3_, and intracellular calcium levels were measured. *D* and *E*, HCT116 cells were transfected with 5 μg of RGS1-HA or 10 μg of RGS2-HA, followed by treatment with 10 μM of CYM5520 (*D*) or CYM5541 (*E*). GST pull-down assays were performed by incubating bacterially expressed GST-Rhotekin-RBD protein–conjugated beads with HCT116 cell lysates. The bound proteins were analyzed by immunoblotting with anti-RhoA antibodies. Ten percent of the cell lysate was loaded as input for the assay, and immunoprecipitation efficiency was confirmed using anti-Rho and anti-HA antibodies. The expression levels of GST-Rhotekin-RBD protein were validated by Coomassie blue staining. ∗*p* < 0.05, ∗∗*p* < 0.01, and ∗∗∗*p* < 0.005, compared with unstimulated control; and #*p* < 0.05, ##*p* < 0.01, and ###*p* < 0.005, compared with agonist-stimulated control. Each experiment was independently repeated at least three times. Data are presented as mean ± SD, and statistical significance was assessed using one-way ANOVA followed by Tukey’s post hoc test. GST, glutathione-*S*-transferase; RGS2, regulator of G protein signaling 2; S1P2, sphingosine-1-phosphate receptor 2; S1P3, sphingosine-1-phosphate receptor 3.

Given that S1P_2_ and S1P_3_ receptors are coupled with Gαi, Gαq, and Gα12 (21), we assessed the impact of RGS2 expression on Gα signaling activated by S1P_2_ and S1P_3_. To specifically examine S1P_2_- and S1P_3_-mediated Gαi signaling, we utilized HCT116 cells stably expressing the CAMYEL plasmid (HCT116-CAMYEL). The cells were transfected with RGS1 or RGS2 and subsequently treated with the specific agonists CYM5520 and CYM5541 (Fig. 4, *A* and *B*). Activation of both receptors resulted in a significant decrease in intracellular cAMP levels, which was further attenuated by RGS2 expression, not by RGS1. These results suggest that RGS2 specifically inhibits S1P_2_- and S1P_3_-medicated Gαi signaling. We next investigated the effect of RGS2 on S1P_2_- and S1P_3_-mediated Gαq signaling by measuring intracellular calcium concentrations. For this, 293T cells were transfected with RGS proteins and treated with S1P as a common agonist for both S1P_2_ and S1P_3_ (Fig. 4*C*). Since S1P_2_ and S1P_3_ are the only S1P receptors coupled to Gαq signaling (21), we used S1P instead of receptor-specific agonists. S1P treatment induced an increase in intracellular calcium levels, which was significantly inhibited by RGS2 expression, but not by RGS1, indicating that RGS2 specifically inhibits S1P_2_- and S1P_3_-mediated Gαq signaling. Furthermore, we assessed whether RGS2 inhibits S1P_2_- and S1P_3_-mediated Gα12 signaling by evaluating RhoA activity. HCT116 cells transfected with RGS proteins were stimulated with an agonist for S1P_2_ and S1P_3_, followed by a RhoA activity assay using cell lysates (Fig. 4, *D* and *E*). Activation of both S1P_2_ and S1P_3_ significantly increased RhoA activity; however, this activation was inhibited by RGS2 expression, indicating that RGS2 suppresses S1P_2_- and S1P_3_-mediated Gα12 signaling. In contrast, RGS1 did not affect S1P receptor–mediated RhoA activation. Collectively, RGS2 attenuates all tested Gα signaling pathways coupled to S1P_2_ and S1P_3_.

### Inhibitory effects of RGS2 on S1P_2_- and S1P_3_-mediated cell migration in HCT116 cells

**Figure 5:**
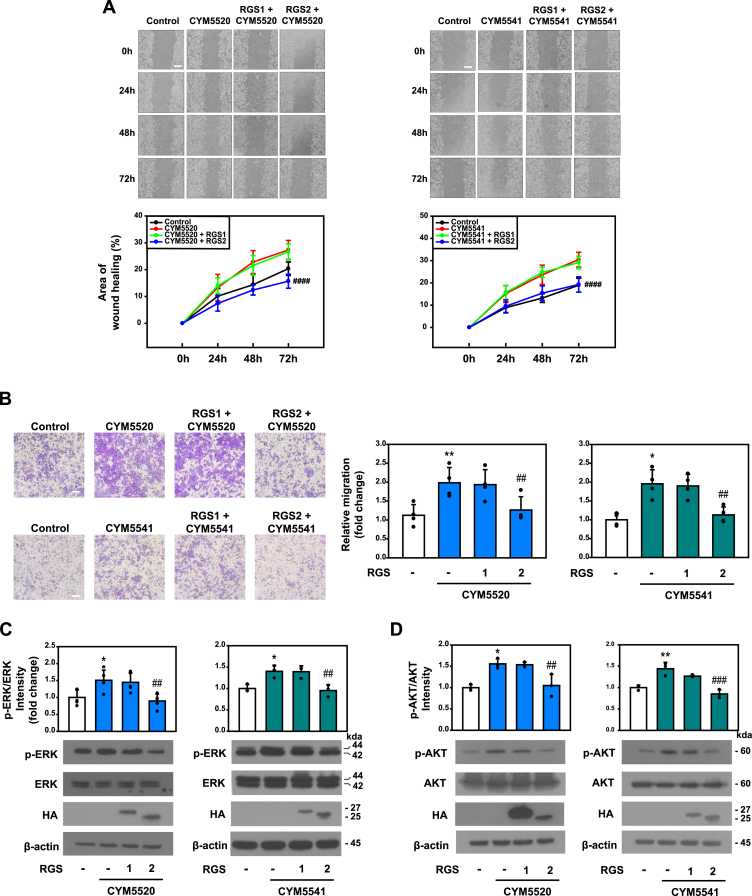
**Inhibitory effects of RGS2 on cell migration mediated by S1P2 or S1P3.***A*, HCT116 cells transfected with RGS proteins were treated with 10 μM of CYM5520 (S1P_2_ agonist) or CYM5541 (S1P_3_ agonist). Scratches were introduced into the cell monolayers, and the cells were observed under an optical microscope at 0, 24, 48, and 72 h. Scale bar represents 500 μm. *B*, HCT116 cells were transfected with RGS proteins and subsequently treated with 10 μM of CYM5520 or CYM5541. After incubation for 6 h, the cells were stained with crystal violet, and migrated cells were visualized under an optical microscope. Scale bar represents 100 μm. *C* and *D*, HCT116 cells were transfected with RGS proteins and treated with 10 μM of the agonists (CYM5520 or CYM5541) for 10 min (for ERK and p-ERK) (*C*) or 5 min (for AKT and p-AKT) (*D*). Immunoblot analysis was performed using antibodies against ERK, p-ERK, AKT, and p-AKT. Relative band intensities of p-ERK/ERK and p-AKT/AKT were quantified using ImageJ software. ∗*p* < 0.05, ∗∗*p* < 0.01 compared with unstimulated control; and ##*p* < 0.01, ###*p* < 0.005, and ####*p* < 0.001, compared with the CYM5520- or CYM5541-stimulated control. Data are presented as mean ± SD and are representative of at least three independent experiments. Statistical significance was determined using one-way ANOVA followed by Tukey’s post hoc test. ERK, extracellular signal–regulated kinase; RGS2, regulator of G protein signaling 2; S1P2, sphingosine-1-phosphate receptor 2; S1P3, sphingosine-1-phosphate receptor 3.

We examined whether RGS2 inhibits S1P_2_- and S1P_3_-mediated cell migration. Wound healing assays using HCT116 cells expressing RGS proteins were performed in the presence of specific agonists (Fig. 5*A*). Quantitative analysis of the healing area showed that stimulation with S1P_2_ and S1P_3_ agonists significantly induced cell migration, which was markedly suppressed by RGS2 expression. In contrast, RGS1 expression had no effect on agonist-stimulated cell migration. To further validate these findings, transwell assays were performed, and the results were quantified (Fig. 5*B*). Consistent with the wound healing assay results, S1P_2_- and S1P_3_-mediated cell migration was significantly inhibited by RGS2 expression, whereas RGS1 expression showed no inhibitory effect. We next investigated the molecular mechanisms underlying the inhibitory effect of RGS2 on S1P_2_- and S1P_3_-stimulated cell migration. Phosphorylation of extracellular signal–regulated kinase (ERK) and AKT is known to be essential for promoting cell migration in cancer cells (22, 23). HCT116 cells were transfected with either RGS1 or RGS2 and stimulated with CYM5520 or CYM5541. Cell lysates were analyzed by immunoblotting using antibodies against phosphorylated ERK and AKT, and band intensities were quantified. ERK phosphorylation was significantly induced by treatment with S1P_2_ and S1P_3_ agonists, but this induction was markedly inhibited by RGS2 expression, whereas RGS1 had no effect (Fig. 5*C*). Similar to the results observed for phospho-ERK, AKT phosphorylation induced by S1P_2_ and S1P_3_ activation was significantly downregulated by RGS2 but not by RGS1 (Fig. 5*D*). Collectively, these findings demonstrate that RGS2 inhibits S1P_2_- and S1P_3_-mediated cell migration by suppressing ERK and AKT phosphorylation.

To further investigate whether ERK and AKT phosphorylation are functionally involved in S1P_2_- and S1P_3_-mediated cell migration, HCT116 cells were pretreated with the ERK inhibitor PD98059 or the AKT inhibitor MK2206 prior to stimulation with CYM5520 or CYM5541. Wound healing assays revealed that inhibition of either ERK or AKT markedly reduced S1P_2_- and S1P_3_-induced cell migration. Consistent with this, immunoblot analysis confirmed that the phosphorylation of ERK and AKT was effectively suppressed by the respective inhibitors. These findings suggest that S1P_2_- and S1P_3_-mediated migration of HCT116 cells is critically dependent on ERK and AKT signaling pathways (Supplementary Information 4).

In our experimental system, RGS1 was initially utilized as a negative control. However, RGS1 consistently exhibited no apparent effects in multiple functional assays. To rule out the possibility that this lack of response was due to a nonfunctional construct, we conducted additional validation experiments to assess its biological activity. Previous studies have reported that RGS1 contributes to melanoma progression (24, 25). To evaluate whether the RGS1 construct used in our study retained functional activity, we assessed its effects on cell migration and downstream signaling pathways in melanoma cells. Wound healing assays demonstrated that RGS1 significantly enhanced melanoma cell motility, and immunoblot analysis revealed increased phosphorylation of ERK and AKT following RGS1 expression (Supplementary Information 5). In contrast, RGS2 expression had no effect on either cell migration or ERK activation under the same conditions. These findings confirm the functional integrity of the RGS1 construct and support its ability to activate promigratory signaling pathways in melanoma cells.

### Suppression of S1P_2_- and S1P_3_-induced cancer-related gene expression by RGS2

**Figure 6:**
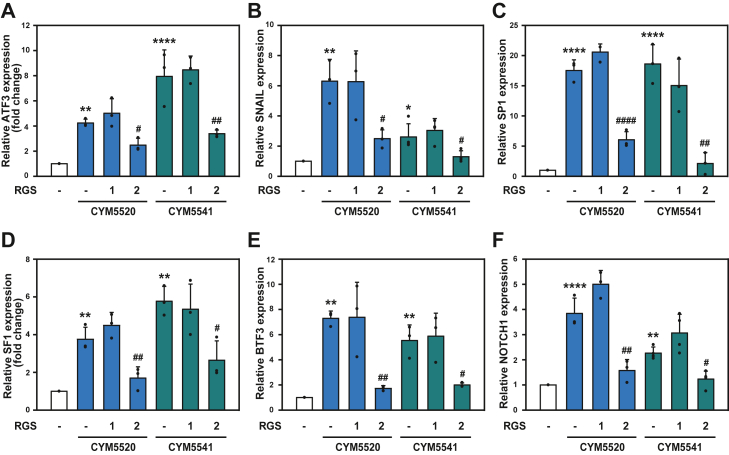
**Inhibitory effects of RGS2 expression on S1P2- and S1P3-mediated cancer progression–related gene expression.***A*–*F*, HCT116 cells were transfected with 2 μg of either RGS1-HA or RGS2-HA expression plasmids. After transfection, cells were treated with 10 μM of either CYM5520 (S1P_2_ agonist) or CYM5541 (S1P_3_ agonist) for 6 h. The relative expression levels of cancer progression–related genes, including ATF3 (*A*), SNAIL (*B*), SP1 (*C*), SF1 (*D*), BTF3 (*E*), and NOTCH1 (*F*), were quantified using RT–qPCR. Gene expression levels were normalized to GAPDH as an internal control. ∗*p* < 0.05, ∗∗*p* < 0.01, and ∗∗∗∗*p* < 0.001, compared with unstimulated control; and #*p* < 0.05, ##*p* < 0.01, and ####*p* < 0.001, compared with CYM5520- or CYM5541-stimulated control. The results are presented as mean ± SD and are representative of at least three independent experiments, with statistical significance assessed using one-way ANOVA followed by Tukey’s post hoc test. qPCR, quantitative PCR; RGS2, regulator of G protein signaling 2; S1P2, sphingosine-1-phosphate receptor 2; S1P3, sphingosine-1-phosphate receptor 3.

Our previous data demonstrated that RGS2 inhibits S1P_2_- and S1P_3_-mediated cell migration in HCT116 cells. To further explore this mechanism, we examined whether RGS2 suppresses S1P receptor–mediated expression of tumor-related genes. Primer sets for several genes, including ATF3, SNAIL, SP1, SF1, BTF3, and NOTCH1, were designed (Table 1). HCT116 cells were transfected with either RGS1 or RGS2 and stimulated with CYM5520 or CYM5541, as indicated. Total RNA was extracted, and RT–qPCR analysis was performed to quantify the expression levels of the target genes (Fig. 6, *A*–*F*). The results revealed that RGS2 significantly suppressed agonist-induced expression of all tested genes, highlighting its inhibitory role in S1P_2_- and S1P_3_-mediated cancer-related gene expression in HCT116 cells.

**Table 1:** Primer sets

Gene	Forward primer	Reverse primer	Annealing temperature (°C)
ATF3	5′-CTG CAG AAA GAG TCG GAG-3′	5′-TGA GCC CGG ACA ATA CAC-3′	48
SNAIL	5′-GAA AGG CCT TCA ACT GCA AA-3′	5′-TGA CAT CTG AGT GGG TCT GG-3′	50
SP1	5′-GCCTCC AGA CCA TTA ACC TCA GT-3′	5′-GCT CCA TGA TCA CCT GGG GCA T-3′	56
SF1	5′-GCA TCT TGG GCT GCC TGC AG-3′	5′-CCT TGC CGT GCT GGA CCT GG-3′	58
BTF3	5′-AGC TTG GTG CGG ATA GTC TGA-3′	5′-GTG CTT TTC CAT CCA CAG ATT G-3′	52
NOTCH1	5′-AAG CTG CAT CCA GAG GCA AAC-3′	5′-TGG CAT ACA CAC TCC GAG AAC AC-3′	54
S1P_2_	5′-ACC ATG GGC AGC TTG TAC TC-3′	5′-AAT GGC GCA ACA GAG GAT GA-3′	51
S1P_3_	5′-AGG CTC AGT GGT TCA TCG TG-3′	5′-GTT GCA GAC CAG ACG GAA GA-3′	52
GAPDH	5′-TGGGCT ACA CTG AGC ACC AG-3′	5′-GGG TGT CGC TGT TGA AGT CA-3′	52

## Discussion

In this study, we demonstrated that RGS2 specifically interacts with S1P_2_ and S1P_3_ in live cells, leading to inhibition of receptor-mediated Gα signaling pathways, cell migration, and cancer-related gene expression. These findings highlight the pivotal role of RGS2 in regulating S1P_2_- and S1P_3_-mediated signaling, which underlie cancer progression–related phenotypes.

RGS proteins are well known for interacting with Gα subunits and suppressing their activity. In addition, they are reported to inhibit GPCR activity through direct or indirect interactions. Notably, the third intracellular loop (i3) of the β2-adrenergic receptor directly interacts with RGS2, leading to the regulation of Gαs-dependent signaling (26). Similarly, the α1A-adrenergic receptor also binds to i3 of RGS2, resulting in the inhibition of Gαq-mediated signaling. This interaction further induces the translocation of RGS2 from the cytoplasm to the cell membrane (27). In addition, RGS2 interacts with the M1 muscarinic acetylcholine receptor through i3 domain and inhibits Gαq-mediated signaling. Interestingly, the M1 receptor forms a ternary complex with Gα and RGS2, enhancing its regulatory effects on signaling pathways (28). Our previous studies demonstrated that RGS2 specifically interacts with type 1, 2, and 4 PARs and inhibits Gα signaling pathways (15, 16, 29). Notably, RGS2 suppresses PAR4-mediated cell proliferation and the expression of cancer-related genes (16). GPR55 also associates with RGS2, and its signaling is attenuated by RGS2 expression (17). In this study, we report a novel finding that, similar to the previously mentioned GPCRs, S1P_2_ and S1P_3_ interact with RGS2, leading to the suppression of GPCR-mediated Gα signaling. Although not experimentally confirmed in this study, the i3 domain of GPCRs is recognized as a critical region for interacting with RGS domains. Therefore, it is hypothesized that the i3 domains of S1P_2_ and S1P_3_ play a pivotal role in their interaction with RGS2.

Previous studies have shown that interactions between GPCRs and RGS2 proteins often involve Gα subunits, forming a ternary complex that suppresses Gα-mediated signaling (14, 15). Consistent with these findings, our BRET analysis in 293T and HCT116 cells demonstrated that Gα participates in the interactions of S1P_2_ and S1P_3_ with RGS2. We propose that RGS2 forms a ternary complex with S1P_2/3_ and Gα at the plasma membrane, thereby inhibiting S1P_2/3_-mediated signaling. This hypothesis is further supported by our observation that RGS2 translocates to the plasma membrane in the presence of S1P_2_ and S1P_3_. Furthermore, the interactions among S1P receptors, RGS2 and Gα, were found to remain intact even after agonist stimulation of S1P_2_ and S1P_3_. These findings lead us to speculate that RGS2 inhibits Gα function by preventing the dissociation of Gα from S1P_2/3_ when localized at the plasma membrane.

In the BRET analysis shown in Figure 1, the interaction between the receptors and RGS2 remained consistent regardless of the type of Gα subunit coexpressed. This observation may reflect the contribution of endogenous Gα proteins that are already sufficient to stabilize the receptor–RGS2 interaction, even in the presence of exogenous Gα isoforms. To better clarify the mechanistic basis of these interactions, future studies using Gα-deficient cell systems or RGS2 mutants impaired in G protein binding would be highly informative.

RGS proteins are widely recognized as GTPase-activating proteins, but they have also been shown to regulate cell migration and growth in cancer cells (30, 31). RGS2, in particular, is highly expressed in normal human cells but is significantly downregulated in cancer cells (32). These findings indicate that RGS2 plays a crucial regulatory role in modulating cancer-related cellular processes, in part through its ability to regulate key signaling pathways. Among these pathways, the phosphorylation of ERK and AKT plays a pivotal role in cancer progression–associated signaling events. RGS2 has been shown to suppress oncogenic signaling by inhibiting the phosphorylation of ERK and AKT (19, 33) This mechanism is particularly evident in colon tumors, where ERK and AKT phosphorylation are critical regulatory events driving malignant transformation and cancer cell behavior (34, 35).

S1P receptors are implicated in the progression of various tumor types. S1P signaling influences tumor growth, metastasis, and chemoresistance, making it a significant target in cancer research (36). In addition, S1P receptors play crucial roles in tumor angiogenesis and immune cell trafficking, further contributing to cancer progression (37). Among S1P receptors, both S1P_2_ and S1P_3_ have been associated with cancer-related cellular processes in multiple tumor types, including colon cancer (20). Our study demonstrates that activation of S1P_2_ and S1P_3_ significantly increases cell migration and cancer-related gene expression through ERK and AKT phosphorylation, and these effects were notably downregulated by RGS2. We propose that RGS2 suppresses Gα signaling triggered by S1P_2_ and S1P_3_ activation, subsequently reducing ERK and AKT phosphorylation. This suppression likely leads to decreased migration of HCT116 cells, highlighting the potential of RGS2 as a key regulator of S1P-mediated oncogenic signaling. We hypothesize that Gα is the primary target of RGS2; however, we cannot exclude the possibility of its involvement at downstream signaling events. The pathway includes numerous regulatory molecules, making it difficult to identify the precise site of the action of RGS2.

Although the enhanced wound closure observed in our assays may raise the possibility of increased cell proliferation contributing to the migratory phenotype, our long-term proliferation analysis revealed no significant difference between agonist-treated and control cells. This suggests that the observed increase in wound closure is unlikely to be due to proliferative effects but rather reflects a genuine enhancement of cell motility in response to S1P_2_ and S1P_3_ activation.

ATF3 has been implicated in the progression of colon cancer, serving as a promoter in this process. For instance, ATF3 enhances colon cancer metastasis by increasing cell motility and invasion (38). In addition, ATF3 is highly expressed in colon cancer cells, and its overexpression is associated with an increased invasive phenotype, further supporting its role in driving cancer progression and metastasis (39). SNAIL is another key transcription factor involved in colon cancer progression. Overexpression of SNAIL promotes colon cancer progression, whereas its knockdown significantly reduces cancer growth and metastasis (40). Furthermore, SNAIL contributes to cell growth, migration, and epithelial–mesenchymal transition (EMT) both *in vitro* and *in vivo* (41). SP1 has been linked to oncogenic processes in colon cancer. It promotes the transcription of FoxO3a, a cancer-related gene, by binding to its promoter region. This upregulation of FoxO3a enhances tumor volume and progression, highlighting the role of SP1 in cancer growth (42). SF1 is implicated in the regulation of Wnt signaling in colon cancer cells, particularly in the SW480 cell line. This regulatory role suggests that SF1 may influence developmental pathways and functions critical to cancer progression (43). BTF3 is another factor contributing to colon cancer progression. Knockdown of BTF3 suppresses cell proliferation, whereas its overexpression enhances cell migration, emphasizing its role in promoting protumorigenic phenotypes in colon cancer cells (44). Moreover, BTF3 is overexpressed in cancer cells and promotes EMT, further driving cancer progression (45). NOTCH signaling is overexpressed in colon cancer and plays a critical role in tumor initiation and progression (46). Specifically, NOTCH1 expression is inversely correlated with survival rates in colon cancer patients, underscoring its significance as a prognostic marker (47). In this study, we primarily focused on analyzing the role of RGS2 in colon cancer–associated cellular phenotypes, particularly in terms of cell migration and the expression of cancer-related genes. However, our findings indicate that RGS2 not only suppresses the expression of genes involved in cell migration but also reduces the expression of genes associated with cell growth, cancer cell invasion, and EMT. These findings suggest that RGS2 may regulate multiple pathways involved in colon cancer progression. Therefore, further investigations into aspects beyond cell migration are warranted to fully elucidate the role of RGS2 in colon cancer.

We used RGS1 as a negative control to investigate the regulatory activity of RGS2 in suppressing S1P_2_- and S1P_3_-mediated signaling. Unlike RGS2, which interacts with S1P_2_ and S1P_3_ receptors, RGS1 does not bind to these receptors and does not influence their signaling pathways, making it a suitable negative control for this study. However, several studies have shown that RGS1 interacts with other receptors (48) and may play a role in inhibiting cancer cell progression. For example, in esophageal cancer, microRNA-mediated downregulation of RGS1 has been associated with increased cell proliferation, migration, and the expression of cancer-related genes (49). Similarly, in gastric cancer, RGS1 knockdown has been reported to reduce cell migration and growth (50). These findings indicate that RGS1 may be functionally relevant to oncogenic processes in certain cancer types. Despite these observations, RGS1 remains an appropriate choice as a negative control in this study for two reasons. First, the role of RGS1 in colon cancer–associated phenotypes is not well documented. Unlike its established roles in esophageal and gastric cancers, there is limited evidence linking RGS1 to colon cancer progression. Second, while RGS1 is known to interact with other receptors, it does not interact with S1P_2_ or S1P_3_, making it functionally distinct in this context. Furthermore, our previous research supports the use of RGS1 as a negative control (17). In HCT116 colon cancer cells, RGS2 was shown to interact with GPR55, effectively suppressing GPR55-mediated oncogenic signaling. In contrast, RGS1 did not exhibit such interactions or effects. This distinction highlights RGS1’s suitability as a negative control for exploring the relationship between RGS proteins and cancer-associated signaling events in colon cancer cells.

In this study, we elucidated the roles of S1P_2_ and S1P_3_, along with the function of RGS2, in colon cancer cells. Our findings demonstrate that the activation of S1P_2_ and S1P_3_ promotes cancer-associated cellular phenotypes, whereas RGS2 suppresses this activity through direct interactions with these receptors. These results suggest that RGS2 holds potential as a therapeutic target for mitigating S1P_2_- and S1P_3_-mediated oncogenic signaling. Further studies are warranted to explore the precise mechanisms underlying these interactions and to evaluate the therapeutic efficacy of targeting RGS2 *in vivo*. Such investigations could pave the way for novel therapeutic strategies against colon cancer.

## Experimental procedures

### Plasmid DNA constructs

The mCherry-tagged S1P receptor constructs (mCherry-S1P_2_ and mCherry-S1P_3_) were generated by digesting pVenus-N1-S1P_2_ and pVenus-N1-S1P_3_ with HindIII and KpnI restriction enzymes to obtain S1P_2_ and S1P_3_ fragments, which were subsequently inserted into the pmCherry-N1 vector digested with the same enzymes. The membrane-anchored YFP construct (Lyn-YFP-FRB1N) was obtained from Addgene. The RGS2^S179D^ mutant was generated by site-directed mutagenesis, commissioned to Bioneer Corporation.

### Cell culture and transfection

The 293T (human embryonic kidney), A375 (human melanoma), and HCT116 (human colorectal carcinoma) cell lines were obtained from the Korean Cell Line Bank and cultured at 37 °C in a humidified incubator with 5% CO_2_. 293T and A375 cells were maintained in Dulbecco’s modified Eagle’s medium supplemented with 100 U/ml penicillin, 100 μg/ml streptomycin, and 10% fetal bovine serum, whereas HCT116 cells were cultured in Dutch-modified RPMI1640 medium supplemented with 100 U/ml penicillin, 100 μg/ml streptomycin, and 10% fetal bovine serum (FBS). For transfection, plasmid DNA and polyethyleneimine (Polysciences, Inc) were mixed at a 1:3 ratio (w/w) and incubated at room temperature for 15 min to form DNA–polyethyleneimine complexes. The transfection complexes were subsequently added to cells maintained in culture medium containing 5% FBS, followed by incubation for 24 h at 37 °C in a humidified atmosphere with 5% CO_2_.

### BRET analysis

293T and HCT116 cells were seeded into 6-well tissue culture plates at a density of 3.5 × 10^5^ cells per well and incubated for 24 h prior to transfection. The cells were then transfected with BRET donor plasmids along with acceptor plasmids, as indicated. To maintain a consistent total DNA amount, an empty vector (pcDNA3.1) was added as necessary. After 24 h of transfection, cells were washed once with PBS and harvested in Tyrode’s solution (140 mM NaCl, 5 mM KCl, 1 mM MgCl_2_, 1 mM CaCl_2_, 0.37 mM NaH_2_PO_4_, 24 mM NaHCO_3_, 10 mM Hepes, and 0.1% glucose, pH 7.4). The harvested cells were transferred to gray 96-well plates (OptiPlates; PerkinElmer Life Sciences). Expression of the acceptor was measured using a VICTOR X2 multilabel plate reader (PerkinElmer Life Sciences) equipped with a 485 nm excitation and 530 nm emission filter. BRET signals were recorded following a 2 min incubation with the luciferase substrate, coelenterazine H (5 μM; Nanolight Technologies). Fluorescence signals (530 ± 20 nm) and luciferase signals (480 ± 20 nm) were measured to calculate the BRET ratio. The agonist was preincubated with the cells for 3 min before the addition of the substrate. The BRET ratio was calculated as the ratio of light emitted from luciferase to the fluorescence signal, and background BRET ratios were subtracted to determine the net BRET values. All measurements were performed in six replicates.

### Coimmunoprecipitation

293T and HCT116 cells were seeded at a density of 1.0 × 10^6^ cells per 100-mm tissue culture dish and incubated for 24 h. 293T cells were transfected with the appropriate plasmids and further cultured for an additional 24 h. In contrast, HCT116 cells were not transfected; cell lysates were prepared directly after the initial 24-h incubation. Cells were then washed with PBS and lysed using a 1% PBTX solution (PBS containing 1% Triton X-100) supplemented with protease inhibitors. One milligram of cell lysate was precleared by incubating with 10 μl of Protein A-Sepharose beads (Sigma–Aldrich) for 1 h at 4 °C with rotation. The lysate was then incubated with 1 μg of antibodies against GFP (ABclonal; catalog number: AE078), S1P_2_ (ABclonal; catalog number: A 18205), or S1P_3_ (ABclonal; catalog number: A15664) at 4 °C for 16 h. Subsequently, 50 μl of Protein A-Sepharose CL-4B beads (Sigma–Aldrich) were added and incubated with rotation at 4 °C for 4 h. The bead complexes were pelleted by centrifugation at 848*g* for 10 s at 4 °C and washed five times with lysis buffer. Bound proteins were eluted with 2X SDS sample buffer and analyzed by Western blotting. Proteins were resolved on an 8 to 12% SDS-PAGE gel and transferred onto a polyvinylidene difluoride membrane. To block nonspecific binding, the membrane was incubated with 5% nonfat dry milk in blocking buffer for 1 h at room temperature. The membrane was then incubated overnight at 4 °C with primary antibodies, including Renilla luciferase antibodies (1:500 dilution; Sigma–Aldrich, catalog number: MAB4400), anti-GFP monoclonal antibodies (1:1000 dilution), anti-RGS1 polyclonal antibodies (1:1000 dilution; Abnova, catalog number: H00005996-B02P), and RGS2 monoclonal antibodies (1:1000 dilution; Abnova, catalog number: H00005997-M01). For detection, horseradish peroxidase–conjugated secondary antibodies were used: anti-rabbit immunoglobulin G (1:5000 dilution; Invitrogen, catalog number: G21234) and anti-mouse immunoglobulin G (1:5000 dilution; Cell Signaling Technology, catalog number: 7076S), incubated for 1 h at room temperature. Protein visualization was performed using an ECL kit (Elpis-Biotech, Inc), following the manufacturer’s instructions.

### Confocal microscopy imaging

293T cells were seeded onto coverslips precoated with poly-d-lysine (50 μg/ml). Cells were transfected with plasmids encoding S1P_2_-mCherry, S1P_3_-mCherry, RGS1-Venus, and RGS2-Venus, as indicated. After 24 h, the cells were washed once with PBS and fixed with 4% paraformaldehyde in PBS at room temperature for 15 min. Fixed cells were washed twice with PBS. The coverslips were mounted onto glass slides using VECTASHIELD Antifade Mounting Medium (Vector Laboratories). Confocal imaging was performed using a Zeiss LSM 700 laser scanning confocal microscope (Carl Zeiss), equipped with appropriate filters for mCherry (excitation: 555 nm) and Venus (excitation: 488 nm). For quantitative analysis, four square-shaped regions of interest were defined per cell at the cell membrane. The Pearson's correlation coefficient was calculated between the image stacks of the mCherry and Venus channels using ImageJ software (National Institutes of Health).

### CAMYEL assay

To establish HCT116 cells stably expressing the CAMYEL plasmid (HCT116-CAMYEL), HCT116 cells were transfected with the CAMYEL plasmid and incubated for 24 h. Following transfection, G418 (Sigma–Aldrich) was applied to the culture medium for 2 weeks to select for stably expressing cells. For experiments, HCT116-CAMYEL cells were seeded at a density of 7.0 × 10^5^ cells per well in 6-well plates and incubated for 24 h. After incubation, the cells were transfected with indicated plasmids. Following 24 h of incubation, the medium was removed, and cells were washed once with prewarmed Hank's balanced salt solution. Subsequently, 700 μl of prewarmed Hank's balanced salt solution was added to each well, and the cells were collected. The collected cells were transferred to a 96-well plate at 90 μl per well and incubated at 37 °C for 30 min. Fluorescence intensity was measured using a VICTOR X2 multilabel plate reader with 490 nm excitation and 525 nm emission filters at various time points. Changes in cAMP levels were quantified by calculating the ratio of luciferase emission to enhanced YFP emission, expressed as a percentage relative to the control group. To assess the effects of agonists, the S1P_2_ agonist CYM5520 and the S1P_3_ agonist CYM5541 (both Cayman Chemical) were applied at a final concentration of 1 μM immediately before luminescence measurement, following the addition of the luciferase substrate.

### Measurement of calcium mobilization

293T cells were seeded at a density of 7.0 × 10^4^ cells per well in black 96-well plates and incubated for 24 h. The cells were transfected with the indicated plasmids and cultured for an additional 24 h. Subsequently, the medium was replaced with serum-free medium, and the cells were incubated for another 24 h. For the assay, 100 μl of Fluo-4 solution was added to each well, and the cells were incubated for 1 h. The solution was then replaced with 100 μl of Tyrode’s solution. Intracellular calcium mobilization was measured using a VICTOR X2 multilabel plate reader (PerkinElmer Life Sciences) with 490 nm excitation and 525 nm emission filters. Measurements were taken every 10 s over a 2000 s period. The initial fluorescence intensity was designated as F_0_, and subsequent fluorescence intensities were recorded as F. The relative fluorescence change (ΔF/F_0_) was calculated and expressed as a percentage of the baseline intensity (F_0_). S1P was applied at a final concentration of 30 μM immediately before measurement.

### Measurement of RhoA activation

BL21 bacterial cells expressing GST-Rhotekin-RBD fusion protein were induced with 0.1 mM isopropyl-β-d-thiogalactoside and lysed in 1% PBTX. The lysate was incubated with glutathione-Sepharose 4B beads (GE Healthcare Life Sciences) in 1% PBTX for 1 h at 4 °C, and the beads were subsequently washed with 1% PBTX. HCT116 cells transfected with indicated plasmids were treated with CYM5520 and CYM5541, at a final concentration of 10 μM for 10 min. Following treatment, the cells were washed with PBS, harvested, and lysed in 1% PBTX supplemented with protease inhibitors. Lysates were incubated with GST-Rhotekin-RBD beads for 4 h at 37 °C. The beads were washed five times with 1% PBTX, and bound proteins were eluted with 2X SDS sample buffer. The eluted proteins were analyzed by immunoblot analysis using antibodies against RhoA (1:500 dilution; Santa Cruz Biotechnology, catalog number: sc-418).

### Wound-healing assay

HCT116 and A375 cells were seeded at a density of 3.5 × 10^5^ cells per well in 6-well plates and transfected with indicated plasmids. When the cells reached approximately 90% confluence, a wound was created in the monolayer using a sterile micropipette tip. The culture medium was then replaced with serum-free medium, and the cells were treated with 10 μM CYM5520 or CYM5541. Wound closure was monitored at 0, 24, 48, and 72 h using an optical microscope. In the case of A375 cells, no agonist treatment was applied following transfection, and wound closure was monitored under the same conditions without pharmacological stimulation. Wound areas were quantified at each time point using ImageJ software, and the percentage of wound closure was calculated relative to the initial wound area.

### Transwell migration assay

HCT116 cells were seeded into 24-well tissue culture plate insert chambers with an 8 μm pore size (VWR International) at a density of 5.0 × 10^4^ cells per well. The lower chambers were filled with serum-free medium. After 24 h, the medium in the insert was replaced with 5% FBS medium, and the lower chambers were replenished with fresh serum-free medium. Cells were transfected with indicated plasmids and incubated for an additional 24 h. Following transfection, the medium in the insert chambers was replaced with serum-free medium containing 10 μM CYM5520 or CYM5541, and the lower chambers were filled with RPMI medium containing 10% FBS. After 6 h of incubation, the cells in the insert chambers were washed with PBS, fixed with paraformaldehyde for 15 min at room temperature, and washed twice with PBS. The fixed cells were then incubated with methanol at room temperature for 10 min and stained with 0.2% crystal violet for 15 min. Excess stain was removed by washing twice with PBS, and nonmigrated cells on the upper side of the membrane were removed using a cotton swab. Migrated cells on the lower side of the membrane were visualized and counted using ImageJ software.

### Measurement of ERK and AKT activation

HCT116 and A375 cells were seeded in 6-well plates at a density of 3.5 × 10^5^ cells per well and cultured for 24 h. The cells were transfected with the indicated plasmids. Following an additional 24 h of incubation, the cells were serum-starved for 24 h in serum-free medium. The cells were treated with 10 μM CYM5520 or CYM5541 for 10 min to assess ERK activation and for 5 min to assess AKT activation. In contrast, A375 cells were processed in parallel without agonist treatment. After washing with PBS, the cells were lysed using radioimmunoprecipitation assay buffer containing protease inhibitors. Protein samples (30 μl) were subjected into immunoblot analysis using the following primary antibodies; ERK (1:1000 dilution; catalog number: 9102S), phospho-ERK (Thr202/Tyr204; 1:1000 dilution; catalog number: 9106S), AKT (1:1000 dilution; catalog number: 9272S), phospho-AKT (Ser473; 1:1000 dilution; catalog number: 9271S) (all from Cell Signaling Technology), and β-actin (1:1000 dilution; Santa Cruz Biotechnology, Inc; catalog number: SC47778).

### RT–qPCR

HCT116 cells were seeded at a density of 3.5 × 10^5^ cells per well in a 6-well culture plate and cultured for 24 h. The cells were transfected with the indicated plasmids. Following transfection, the culture medium was replaced with serum-free medium, and the cells were incubated for an additional 24 h. Subsequently, the cells were treated with either the CYM5520 or CYM5541 at a final concentration of 10 μM for 6 h. Total RNA was extracted using the Easy-Spin Total RNA Extraction kit (Intron Biotechnology) according to the manufacturer’s protocol. Complementary DNA synthesis was performed using AccuPower RT PreMix (Bioneer Corporation) at 42 °C for 1 h. RT–qPCR was performed on the GENECHECKER PCR System (Genesystem) using Rapi:Chip (Genesystem), specific gene primers (Table 1) and Detect Master Mix (Genesystem). The thermocycling conditions were as follows: initial denaturation at 95 °C for 30 s, followed by 50 cycles of 95 °C for 3 s (denaturation), 43 to 56 °C for 3 s (annealing), and 72 °C for 3 s (extension). Gene expression levels were normalized to GAPDH as the reference gene, and relative mRNA expression levels were calculated using the 2^-^ΔΔCq method.

### Statistical analysis

All data are presented as the mean ± SD from at least three independent experiments. Statistical analyses were performed using one-way ANOVA followed by Tukey’s post hoc test to account for multiple comparisons. Data visualization was conducted using SigmaPlot 10.0 (Systat Software Inc.) and GraphPad Prism 8.4.3 (GraphPad Software). Statistical significance was defined as *p* < 0.05, with significance levels indicated as follows: *p* < 0.05 (∗), *p* < 0.01 (∗∗), *p* < 0.005 (∗∗∗), and *p* < 0.001 (∗∗∗∗). *p* Values determined by comparison with a second control group in the graph are denoted with #.

## Data availability

All other data and materials supporting the findings of this study are available from the corresponding author upon reasonable request.

## Supporting information

This article contains supporting information.

## Conflict of interest

The authors declare that they have no conflicts of interest with the contents of this article.
