# Supplementary material for: Regulator of G protein signaling 2 as a suppressor of sphingosine-1-phosphate 2– and 3–mediated signaling in colon cancer cells
Source: J Biol Chem. 2025 Aug 5;301(9):110554. doi: 10.1016/j.jbc.2025.110554 (PMC12405630; doi:10.1016/j.jbc.2025.110554)
Supplement: Supplementary Information 1 [file mmc1.docx]

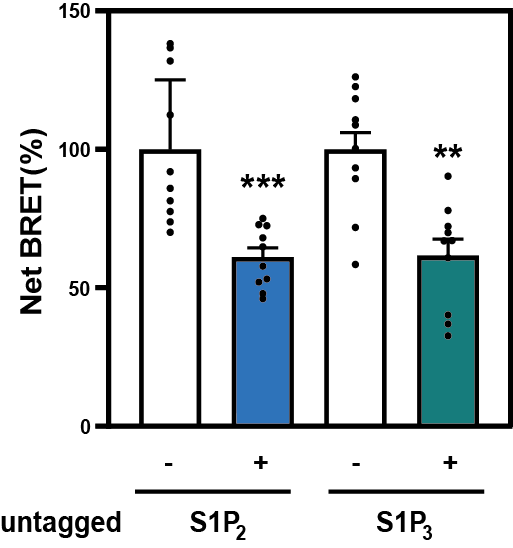


**Supplementary Information 1.** Co-expression of untagged S1P_2_ or S1P_3_ reduces BRET signal with RGS2, supporting the specificity of the interaction. BRET analysis was performed in 293T cells co-transfected with either S1P_2_-Venus (blue, 2.0 μg) or S1P_3_-Venus (green, 2.0 μg) together with RGS2-Luc (0.03 μg), in the absence or presence of untagged S1P_2_ (0.5 μg) or untagged S1P_3_ (0.5 μg), respectively. **P < 0.01, ***P < 0.005 compared to the control. Data represent the mean ± SD of three independent experiments. Statistical analysis was performed using one-way ANOVA followed by Tukey’s post hoc test.
