# Supplementary material for: Regulator of G protein signaling 2 as a suppressor of sphingosine-1-phosphate 2– and 3–mediated signaling in colon cancer cells
Source: J Biol Chem. 2025 Aug 5;301(9):110554. doi: 10.1016/j.jbc.2025.110554 (PMC12405630; doi:10.1016/j.jbc.2025.110554)
Supplement: Supplementary Information 2 [file mmc2.docx]

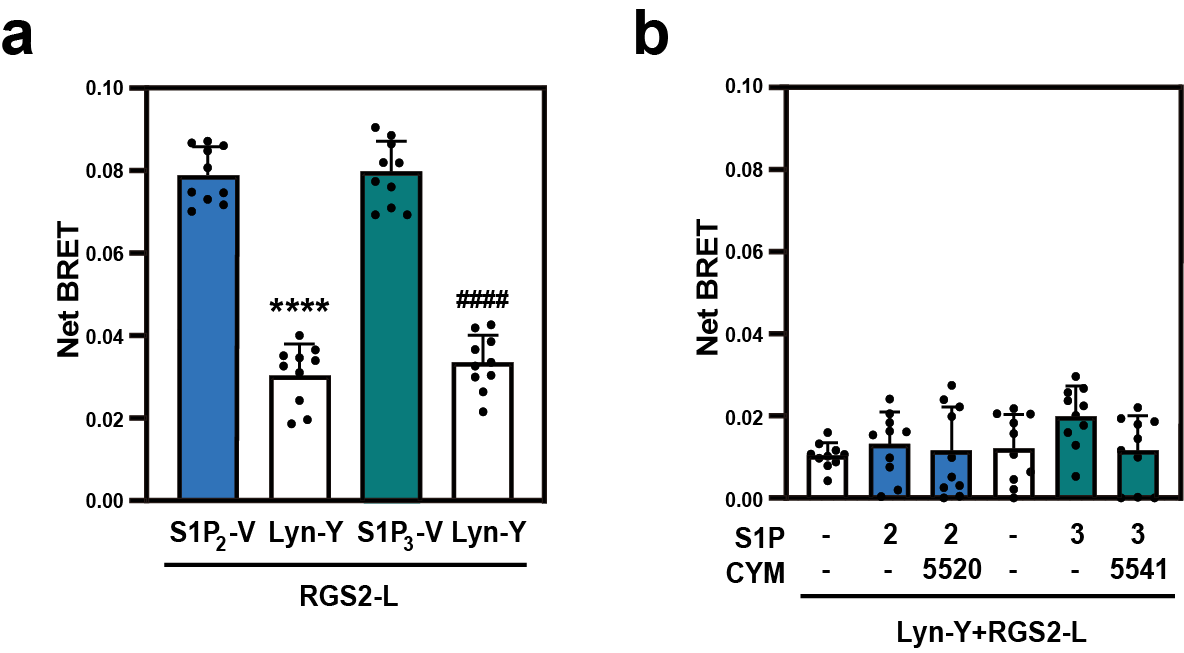


**Supplementary Information 2. Assessment of nonspecific membrane effects and bystander interactions in RGS2–S1P receptor BRET assays.** (a) 293T cells were co-transfected with RGS2-Luc (RGS2-L, 0.03 μg) and either S1P_2_-Venus (S1P_2_-V, 2.0 μg), S1P_3_-Venus (S1P3-V, 2.0 μg), or membrane-anchored Lyn-YFP (Lyn-Y, 2.0 μg), as indicated. Cells were subjected to BRET analysis under basal conditions. ****P < 0.001 vs. S1P_2_-V co-expressed with RGS2-L; ####P < 0.001 vs. S1P_3_-V co-expressed with RGS2-L. (b) 293T cells were co-transfected with RGS2-L (0.03 μg), Lyn-Y (2.0 μg), and either untagged S1P_2_ or S1P_3_ receptors (2.0 μg), as indicated. BRET analysis was performed under basal conditions and following treatment with the S1P_2_-selective agonist CYM5520 or the S1P_3_-selective agonist CYM5541. Data are presented as mean ± SD and are representative of at least three independent experiments. Statistical significance was determined using one-way ANOVA followed by Tukey’s post hoc test.
