# Supplementary material for: Regulator of G protein signaling 2 as a suppressor of sphingosine-1-phosphate 2– and 3–mediated signaling in colon cancer cells
Source: J Biol Chem. 2025 Aug 5;301(9):110554. doi: 10.1016/j.jbc.2025.110554 (PMC12405630; doi:10.1016/j.jbc.2025.110554)
Supplement: Supplementary Information 3 [file mmc3.docx]

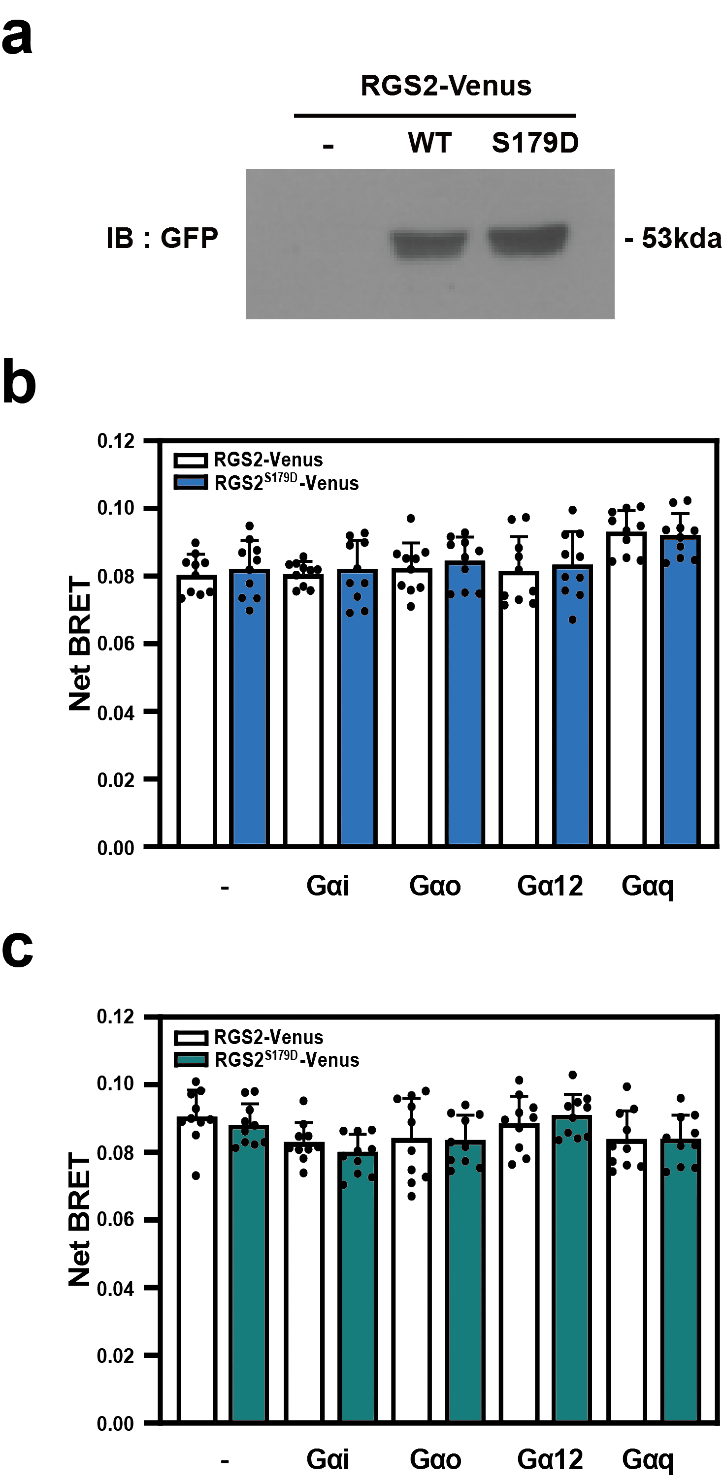


**Supplementary Information 3.** Investigation of the potential involvement of endogenous Gα proteins in the interaction between S1P_2/3_ receptors and RGS2 using an RGS2 mutant. (a) 293T cells were transfected with 2.0 μg of either wild-type (WT) RGS2-Venus or Gαq-binding–deficient RGS2^S179D^-Venus (S179D) plasmids. Protein expression was verified by immunoblotting using an anti-GFP antibody. (b, c) 293T cells were co-transfected with 0.03 μg of S1P_2_-Luc (b) or S1P_3_-Luc (c), 2.0 μg of either wild-type RGS2 or RGS2 ^S179D^-Venus, and additional plasmids encoding various Gα subunits. Data are presented as mean ± SD from at least three independent experiments.
