# Supplementary material for: Regulator of G protein signaling 2 as a suppressor of sphingosine-1-phosphate 2– and 3–mediated signaling in colon cancer cells
Source: J Biol Chem. 2025 Aug 5;301(9):110554. doi: 10.1016/j.jbc.2025.110554 (PMC12405630; doi:10.1016/j.jbc.2025.110554)
Supplement: Supplementary Information 4 [file mmc4.docx]

**
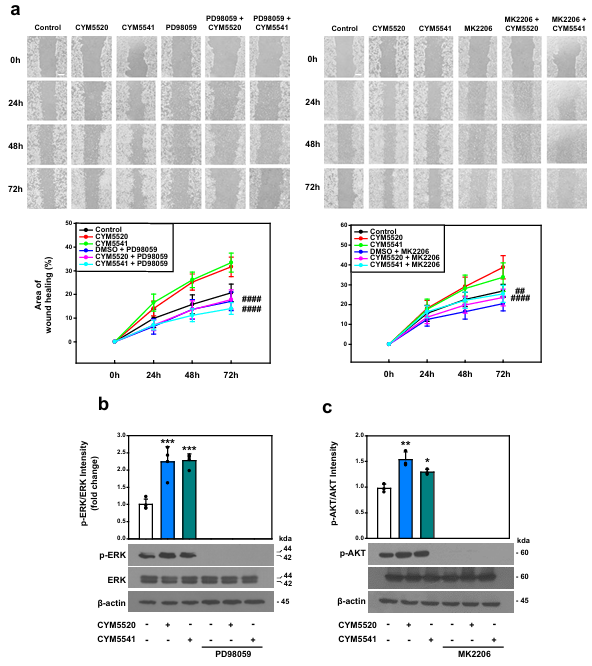
**

**Supplementary Information 4.** Inhibitory effects of ERK or AKT inhibitors on cell migration and intracellular signaling mediated by S1P_2_ or S1P_3_. (a) HCT116 cells were treated with 10 μM of CYM5520 (S1P_2_ agonist) or CYM5541 (S1P_3_ agonist) following a 30 min pretreatment with PD98059 (ERK inhibitor) or MK2206 (AKT inhibitor). Scratches were introduced into the cell monolayers, and cell migration was observed under an optical microscope at 0, 24, 48, and 72 h. Scale bar, 500 μm. ##P < 0.01, ####P < 0.001, compared to the group treated with agonist alone (no inhibitor). (b, c) HCT116 cells were pretreated with PD98059 or MK2206 for 30 min and then stimulated with 10 μM of CYM5520 or CYM5541. Cells were harvested at 10 min (for ERK and p-ERK) or 5 min (for AKT and p-AKT), followed by immunoblot analysis using antibodies against ERK, p-ERK, AKT, and p-AKT. Relative band intensities of p-ERK/ERK and p-AKT/AKT were quantified using ImageJ software. *P < 0.05, **P < 0.01, ***P < 0.005 vs. unstimulated control. The results are representative of at least three independent experiments, and data are presented as mean ± SD. Statistical significance was assessed using one-way ANOVA followed by Tukey’s post hoc test.
