# Supplementary material for: Regulator of G protein signaling 2 as a suppressor of sphingosine-1-phosphate 2– and 3–mediated signaling in colon cancer cells
Source: J Biol Chem. 2025 Aug 5;301(9):110554. doi: 10.1016/j.jbc.2025.110554 (PMC12405630; doi:10.1016/j.jbc.2025.110554)
Supplement: Supplementary Information 5 [file mmc5.docx]

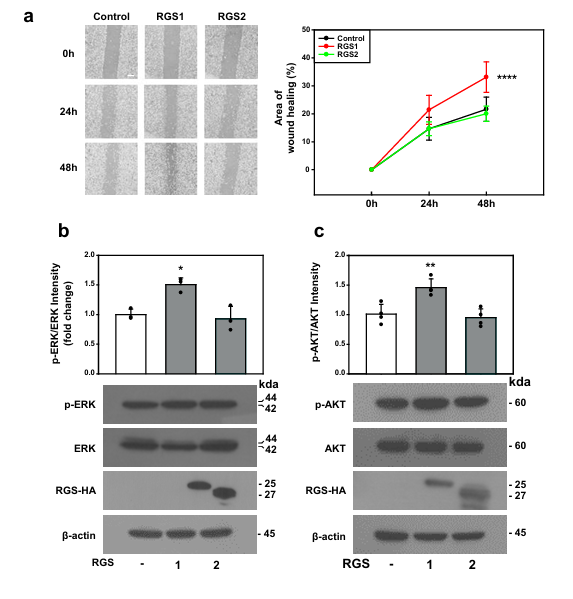


**Supplementary Information 5**. Effects of RGS1 and RGS2 on cell migration and intracellular signaling in A375 cells. (a) A375 cells were transfected with 2.0 μg of either RGS1 or RGS2, and scratches were introduced into confluent cell monolayers. Cell migration was monitored under an optical microscope at 0, 24, and 48 h. Scale bar 500μm. ****P < 0.001, compared to the control group. (b, c) A375 cells were transfected with 2.0 μg of either RGS1 or RGS2, followed by immunoblot analysis using antibodies against ERK, p-ERK, AKT, and p-AKT. Relative band intensities of p-ERK/ERK and p-AKT/AKT were quantified using ImageJ software. *P < 0.05, **P < 0.01, compared to the control group. The results are representative of at least three independent experiments and are presented as mean ± SD. Statistical significance was assessed using one-way ANOVA followed by Tukey’s post hoc test.
